# Integrated Assessment of Inflammatory and Lipid–Metabolic Biomarkers in Psoriasis: Implications for Metabolic Syndrome

**DOI:** 10.3390/life16050821

**Published:** 2026-05-15

**Authors:** Laura-Florina Nistor, Ruxandra Cristina Marin, Delia Mirela Tit, Gabriela S. Bungau, Ada Radu, Timea Claudia Ghitea, Mirela Marioara Toma, Laura Maria Endres

**Affiliations:** 1 Doctoral School of Biomedical Sciences, Faculty of Medicine and Pharmacy, University of Oradea, 410087 Oradea, Romania; cuc.lauraflorina@student.uoradea.ro (L.-F.N.); gbungau@uoradea.ro (G.S.B.); adaradu@uoradea.ro (A.R.); timea.ghitea@csud.uoradea.ro (T.C.G.); lendres@uoradea.ro (L.M.E.); 2 Department of Pharmacology, Clinical Pharmacology and Pharmacotherapy, Faculty of Medicine, Carol Davila University of Medicine and Pharmacy, 050474 Bucharest, Romania; 3 Department of Pharmacy, Faculty of Medicine and Pharmacy, University of Oradea, 410028 Oradea, Romania; toma.mirelamarioara@didactic.uoradea.ro; 4 Department of Psycho-Neurosciences and Recovery, Faculty of Medicine and Pharmacy, University of Oradea, 410073 Oradea, Romania

**Keywords:** psoriasis, inflammation, metabolic syndrome, obesity, comorbidities, predictive modeling

## Abstract

(1) Background: Psoriasis is increasingly recognized as a systemic inflammatory disease associated with metabolic comorbidities. However, the hierarchical relationship between inflammatory activation and insulin resistance in driving metabolic syndrome (MetS) remains incompletely defined. This study aimed to characterize the integrated inflammatory–metabolic architecture of psoriasis using multivariate and latent domain modeling. (2) Methods: In this cross-sectional hospital-based study (2020–2022), 235 adult patients with psoriasis were evaluated. Systemic inflammatory markers (NLR, SII, CRP, ESR) and composite metabolic indices (TyG, AIP, METS-IR) were assessed. Correlation analysis, multivariable linear and logistic regression, interaction modeling, and principal component analysis (PCA) were performed to examine independent associations and underlying domain structure. (3) Results: Inflammatory and metabolic markers showed modest but significant correlations. In multivariable logistic regression, the TyG index was the strongest independent predictor of MetS (OR = 5.15, *p* < 0.001), whereas inflammatory markers did not retain independent significance. An interaction between adiposity and insulin resistance further improved model discrimination (AUC = 0.830). PCA identified two distinct latent domains explaining 69.9% of total variance: an immune–inflammatory domain (NLR, SII, ESR, CRP) and a metabolic–insulin resistance domain (TyG, AIP, METS-IR). Only the metabolic domain independently discriminated MetS. (4) Conclusions: Psoriasis exhibits a multidimensional systemic architecture characterized by partially independent inflammatory and metabolic domains. Although systemic inflammation and metabolic dysfunction coexist, insulin-resistance-related indices were more strongly associated with metabolic syndrome in this cohort.

## 1. Introduction

Psoriasis is a chronic immune-mediated inflammatory disease affecting approximately 4–5% of the global population, with substantial epidemiological burden in Europe and Eastern European countries such as Romania [1,2].

Once considered primarily a dermatologic condition, psoriasis is now widely recognized as a systemic disease characterized by chronic inflammation and multisystem involvement. This systemic perspective reflects the concept of a “psoriatic disease spectrum,” encompassing not only cutaneous manifestations but also cardiometabolic, musculoskeletal, and psychosocial comorbidities [3,4].

Individuals with psoriasis have an increased risk of metabolic syndrome, type 2 diabetes, hypertension, and dyslipidemia, contributing to elevated morbidity and cardiovascular risk [5]. The interaction between immune activation and metabolic dysfunction is central to psoriatic disease. In this context, adipose tissue functions as an active endocrine organ, releasing adipokines and pro-inflammatory mediators that contribute to insulin resistance and systemic inflammation. In parallel, cytokine pathways involving TNF-α, IL-6, and IL-17 further amplify metabolic and vascular dysfunction, together with oxidative-stress-related mechanisms. These interconnected processes highlight the bidirectional relationship between inflammation and metabolic dysregulation in psoriasis [6,7].

Composite inflammatory and metabolic indices have emerged as accessible tools for assessing systemic disease burden in psoriasis. Hematologic markers such as the neutrophil-to-lymphocyte ratio (NLR) and systemic immune–inflammation index (SII) reflect immune activation, while indices such as the triglyceride–glucose (TyG) index, atherogenic index of plasma (AIP), and METS-IR provide surrogate measures of insulin resistance and atherogenic risk [8,9].

At the molecular level, emerging metabolomic and systems biology approaches have revealed widespread metabolic reprogramming in psoriasis. Studies demonstrate dysregulation of amino acid metabolism, lipid pathways, and oxidative stress processes, reflecting profound alterations in cellular energy utilization and immune cell activation. These findings indicate that inflammatory disease is accompanied by systemic metabolic remodeling affecting multiple biochemical networks [10]. Oxidative-stress-related mitochondrial dysfunction is a key mechanism underlying metabolic disturbances across inflammatory conditions, contributing to impaired cellular energetics, increased reactive oxygen species production, and amplification of inflammatory signaling cascades [11]. These metabolic signatures may differentiate disease phenotypes, predict systemic complications, and provide novel targets for therapeutic intervention.

Another emerging area is the phenotypic heterogeneity of psoriasis. While morphological subtypes remain clinically relevant, data-driven approaches (e.g., cluster analysis and principal component analysis) increasingly identify distinct clinico-biological phenotypes. These include metabolically driven disease with obesity and dysglycemia, early-onset high-inflammatory forms, and phenotypes with subclinical systemic inflammation despite limited skin involvement [12].

The growing recognition of psoriasis as a systemic inflammatory–metabolic disorder underscores the need for comprehensive patient characterization beyond skin severity alone. Psoriasis is now widely regarded as a multisystem disease with substantial cardiometabolic involvement, and contemporary expert reviews emphasize routine assessment of systemic comorbidities alongside dermatologic evaluation [13].

Traditional clinical indices, although valuable, may underestimate systemic disease burden and fail to identify individuals at high cardiometabolic risk, highlighting the limitations of morphology-based assessment alone [14]. Integrated assessment of inflammatory and lipid-metabolic biomarkers therefore represents a critical step toward improved risk stratification, early detection of comorbidities, and personalized therapeutic decision-making. Combined evaluation of inflammatory markers and metabolic indices provides more comprehensive characterization of disease activity and cardiometabolic risk than single-domain assessment [15].

However, clinically validated integrative frameworks remain insufficiently standardized and inconsistently applied across real-world settings, and few candidate biomarkers have achieved successful translation into routine clinical practice [13]. Despite strong epidemiologic and pathophysiological links between psoriasis and cardiometabolic disease, clinically actionable integrative frameworks remain insufficiently defined. Surrogate indices of insulin resistance and atherogenic dyslipidemia are increasingly recognized but often evaluated in isolation using heterogeneous methodologies, limiting comparability and translational utility [15]. Similarly, hematologic inflammation composites such as the systemic immune–inflammation index (SII) have been linked to metabolic syndrome in psoriasis, yet their relationship to classical inflammatory markers and insulin resistance surrogates remains incompletely defined within integrative analytical models [14].

Real-world data from Eastern Europe, including Romania, remain limited, with most studies focusing on selected outpatient populations or single comorbidity endpoints rather than comprehensive hospital-based phenotyping integrating inflammatory, metabolic, and patient-centered dimensions [16].

Despite growing recognition of the inflammatory–metabolic interplay in psoriasis, integrative analytical frameworks that simultaneously evaluate inflammatory and metabolic biomarkers within a unified model remain limited, particularly in real-world hospital-based populations.

The present study was designed to investigate the inflammatory–metabolic interface in a hospitalized cohort of patients with psoriasis using an integrated analytical approach combining clinical data and composite laboratory biomarkers of immune activation and insulin resistance. We aimed to characterize inflammatory and metabolic profiles, assess their associations, identify predictors of metabolic syndrome, and explore biomarker structure using multivariable modeling and principal component analysis.

## 2. Results

### 2.1. Baseline Characteristics

A total of 235 adult patients with psoriasis were included in the analysis. The cohort was predominantly middle-aged (median age 58 years), with a balanced sex distribution and similar urban–rural representation. Median disease duration was 10 years.

Most patients had plaque psoriasis (53.2%), while the remainder presented other clinical forms. Psoriatic arthritis was identified in 14.9% of cases. Treatment distribution was heterogeneous, with 43.0% of patients receiving no treatment, 41.3% topical therapy, 26.0% systemic therapy, and a small proportion receiving biologic therapy (4.3%) or phototherapy (4.7%).

Overweight and obesity were highly prevalent, with a median BMI of 28.9 kg/m^2^; more than 80% of patients were above normal weight. Metabolic syndrome was present in 38.7% of the cohort. Cardiometabolic comorbidities were frequent, particularly hypertension (62.1%), diabetes mellitus (27.7%), and cardiovascular disease (31.9%), indicating substantial multimorbidity.

Most patients (90.2%) were hospitalized with psoriasis as a secondary diagnosis, suggesting that admission was primarily driven by comorbid conditions. Detailed demographic and clinical characteristics are presented in Table 1, including psoriasis phenotype, psoriatic arthritis status, and treatment distribution.

### 2.2. Systemic Inflammatory and Metabolic Burden

Systemic inflammatory and metabolic parameters are summarized in Table 2 and Table 3. The cohort exhibited elevated inflammatory activity, with median SII of 668.9, NLR of 2.56, ESR of 21 mm/h, and CRP of 6.8 mg/L. Severe systemic inflammation (SII > 800) was observed in 40.4% of patients, while elevated CRP and ESR were present in 58.3% and 66.8% of cases, respectively.

Metabolic abnormalities were also common, with a high prevalence of dyslipidemia, obesity, and metabolic syndrome (38.7%). Median values of TyG, AIP, and METS-IR were consistent with increased cardiometabolic risk.

When stratified by metabolic syndrome status, patients with MetS showed significantly higher TyG, AIP, and METS-IR values (all *p* < 0.001), with moderate-to-large effect sizes. In contrast, inflammatory markers (SII, NLR, CRP, ESR) did not differ significantly between groups and showed only small effect sizes (Table 4).

### 2.3. Inflammatory–Metabolic Associations

Spearman correlation analysis was performed to evaluate the relationship between systemic inflammatory indices and metabolic dysfunction markers (Table 5).

Acute-phase inflammatory markers showed consistent positive associations with metabolic parameters. Both CRP and ESR showed moderate correlations with TyG, AIP, METS-IR, and triglycerides (all *p* ≤ 0.001).

In contrast, hematological inflammatory indices reflecting innate immune activation showed limited metabolic associations. NLR was not significantly correlated with metabolic indices, while SII showed only a weak association with TyG.

**Figure 1:**
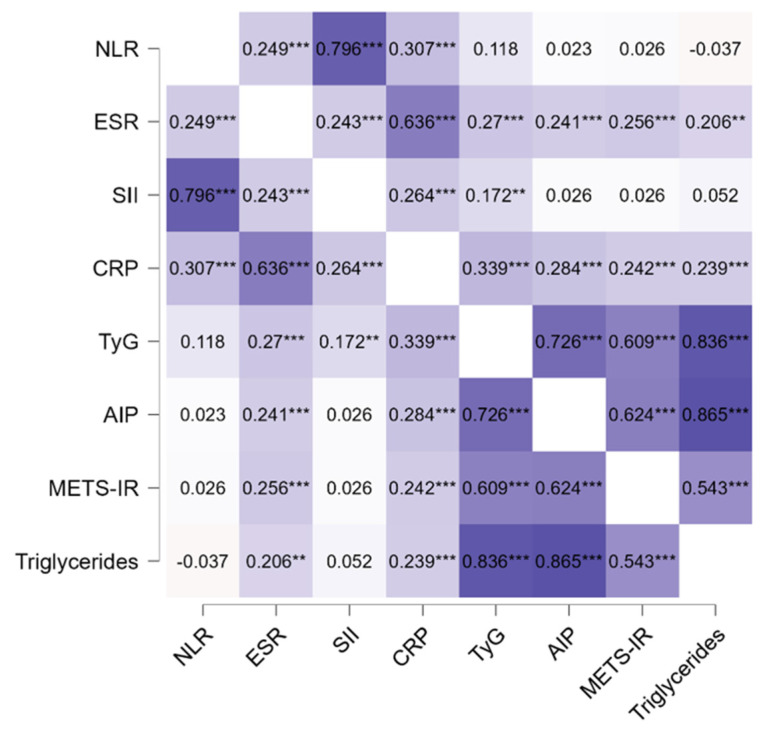
Spearman correlation heatmap of inflammatory and metabolic indices in patients with psoriasis. Color intensity reflects the strength of correlation coefficients (ρ). AIP, atherogenic index of plasma; CRP, C-reactive protein; ESR, erythrocyte sedimentation rate; METS-IR, metabolic score for insulin resistance; NLR, neutrophil-to-lymphocyte ratio; SII, systemic immune–inflammation index; TyG, triglyceride–glucose index; ρ, Spearman correlation coefficient. Statistical significance levels: ** *p* < 0.01, *** *p* < 0.001.

The correlation heatmap (Figure 1) further illustrates this pattern, highlighting a clear separation between hematological markers and metabolic parameters, with CRP and ESR showing consistent positive associations with cardiometabolic indices.

To evaluate the independent contribution of inflammatory markers to insulin resistance, a multivariable linear regression model including CRP, ESR, NLR, and SII was constructed with METS-IR as the dependent variable. The model was statistically significant (R^2^ = 0.054, *p* = 0.015), indicating a modest contribution of systemic inflammation to insulin resistance (Table 6).

Among the included variables, only ESR remained independently associated with METS-IR (β = 0.214, *p* = 0.016), whereas CRP, NLR, and SII were not significant predictors.

These findings suggest that ESR may be more closely associated with metabolic impairment than the other inflammatory indices evaluated in this cohort.

To further evaluate the relative contribution of adiposity and inflammation to insulin resistance, a multivariable linear regression model including age, BMI, CRP, and ESR was constructed. The model demonstrated high explanatory power (R^2^ = 0.767, *p* < 0.001).

BMI showed the strongest independent association with METS-IR (β = 0.856, *p* < 0.001), whereas CRP was only modestly associated (β = 0.086, *p* = 0.041). ESR and age were not significant predictors. These findings suggest that adiposity appears to be more strongly associated with insulin-resistance-related indices than inflammatory markers in this cohort. Disease duration was additionally evaluated as a potential confounder; however, it was not independently associated with METS-IR and did not materially alter the observed associations (*p* = 0.799).

**Figure 2:**
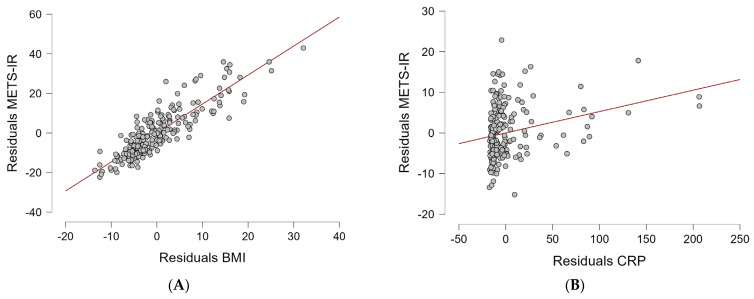
Partial regression plots for the independent associations of BMI (**A**) and CRP (**B**) with METS-IR.

The partial regression plots (Figure 2) illustrate a strong linear relationship between BMI and METS-IR, whereas the association for CRP is comparatively weaker.

Bootstrap validation confirmed coefficient stability, and no relevant multicollinearity was observed (all VIF < 2). These findings indicate that, although systemic inflammation is statistically associated with metabolic impairment, adiposity is more strongly associated with insulin-resistance-related indices in this cohort. The substantial difference in explained variance between the inflammatory-only model (R^2^ = 0.054) and the model including BMI (R^2^ = 0.767) further supports this observation.

### 2.4. Predictors of Metabolic Syndrome

Multivariable logistic regression models were constructed to evaluate independent predictors of MetS and to assess the interaction between adiposity and insulin-resistance-related indices.

In the baseline model including age, BMI, and TyG, the TyG index was strongly associated with metabolic syndrome (OR ≈ 5.0, *p* < 0.001), with good discriminative performance (AUC = 0.801). Age and BMI were also independently associated with MetS (both *p* < 0.001).

To further explore this relationship, centered BMI (BMI_c) and TyG (TyG_c) variables, along with their interaction term, were included in the final model. Model performance improved (AUC = 0.830; Nagelkerke R^2^ = 0.412), and all predictors remained significant.

The interaction term (BMI_c × TyG_c) was independently associated with metabolic syndrome (OR = 1.21, 95% CI 1.07–1.36, *p* = 0.002) (Table 7), indicating that the association between insulin-resistance-related indices and metabolic syndrome increased with higher levels of adiposity.

These findings support an interaction between adiposity and insulin resistance-related indices in relation to metabolic syndrome risk, suggesting a multidimensional metabolic risk profile rather than isolated biomarker effects.

### 2.5. Latent Structure of Systemic Inflammatory and Metabolic Burden

Principal component analysis (PCA) was performed to explore the underlying structure of systemic inflammatory and metabolic biomarkers. Sampling adequacy was acceptable (Kaiser–Meyer–Olkin index = 0.675), and Bartlett’s test of sphericity confirmed the suitability of the data for factor analysis (χ^2^ = 860.941, *p* < 0.001).

**Figure 3:**
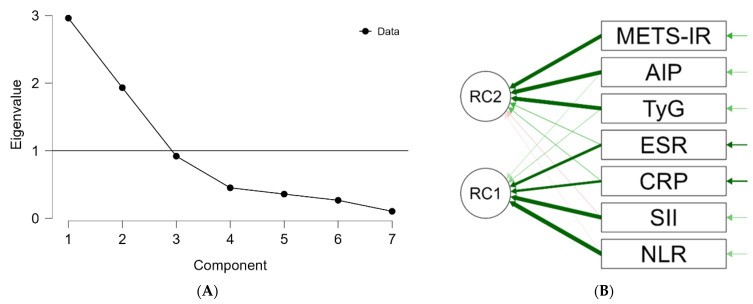
Principal component analysis of inflammatory and metabolic markers in psoriasis: (**A**) Scree plot showing two components with eigenvalues > 1. (**B**) Rotated component structure (Varimax rotation). AIP, atherogenic index of plasma; CRP, C-reactive protein; ESR, erythrocyte sedimentation rate; METS-IR, metabolic score for insulin resistance; NLR, neutrophil-to-lymphocyte ratio; SII, systemic immune–inflammation index; TyG, triglyceride–glucose index. RC1 and RC2 represent the two retained rotated components. Darker arrows indicate stronger variable loadings.

Based on the Kaiser criterion and scree plot inspection (Figure 3A), two components were retained, explaining 69.9% of the total variance. After Varimax rotation, the first component (36.4% variance) was characterized by high loadings of NLR, SII, ESR, and CRP, reflecting an inflammatory domain. The second component (33.5% variance) showed strong loadings for AIP, TyG, and METS-IR, corresponding to a metabolic domain.

The rotated component structure (Figure 3B) demonstrates a clear separation between inflammatory and metabolic markers.

These findings support the structural organization of inflammatory and metabolic biomarkers into distinct domains within the dataset, consistent with the expected clustering of these variables. In line with multivariable analyses, only the metabolic domain showed an independent association with metabolic syndrome, whereas the inflammatory domain did not.

## 3. Discussion

This study provides a comprehensive multidimensional evaluation of inflammatory and metabolic burden in a hospitalized cohort of patients with psoriasis, many of whom were admitted primarily for comorbid conditions. By integrating correlation analysis, multivariable modeling, and principal component analysis, we examined how inflammatory and metabolic biomarkers relate to metabolic syndrome in a high-complexity clinical setting.

The baseline clinical profile reflects substantial cardiometabolic burden, consistent with the systemic nature of psoriatic disease. Patients were predominantly middle-to-older aged, with high prevalence of overweight and obesity, metabolic syndrome, and cardiovascular comorbidities. These findings align with real-world studies reporting metabolic syndrome prevalence of approximately 30–50% among patients with psoriasis, particularly in those with long disease duration and significant comorbidity burden [13].

The hospitalization context further characterizes this cohort as a high-risk population. Most admissions were driven by non-dermatologic conditions, and multimorbidity was frequent, supporting the concept that psoriasis is frequently accompanied by substantial cardiometabolic and multi-organ involvement [17].

Importantly, because the majority of patients had psoriasis recorded as a secondary diagnosis, the cohort likely reflects individuals with substantial comorbidity burden rather than a typical dermatology-based population. This may have influenced the relative prominence of metabolic indices observed in the present study. Accordingly, the predominance of the metabolic domain should be interpreted in the context of a high-risk, comorbidity-driven population and may not fully reflect psoriasis-specific pathophysiology in outpatient settings. Treatment heterogeneity may have also influenced the observed relationships. A substantial proportion of patients were not receiving active therapy, while others were treated with topical, systemic, or biologic agents. Given that systemic and biologic therapies can modulate inflammatory activity and metabolic parameters, this variability may have contributed to the observed dissociation between inflammatory markers and metabolic indices.

Several consistent patterns emerged. Inflammatory and metabolic biomarkers showed modest associations, whereas insulin-resistance-related indices, particularly TyG, demonstrated the strongest independent relationship with metabolic syndrome. Principal component analysis further supported a structured separation between inflammatory and metabolic domains. Together, these findings indicate that while systemic inflammation and metabolic dysfunction frequently coexist in psoriasis, metabolic syndrome in this cohort is more strongly associated with insulin-resistance-related mechanisms than with conventional inflammatory markers [3].

The high baseline cardiometabolic burden of the cohort may have influenced these relationships. In such populations, systemic inflammation may represent a shared background feature, whereas insulin-resistance-related indices better capture the incremental metabolic derangement defining metabolic syndrome [17]. This interpretation is consistent with evidence showing that patients with psoriasis frequently accumulate multiple cardiometabolic comorbidities and may be insufficiently screened for cardiovascular risk, particularly in complex clinical settings [18].

Although inflammation contributes to metabolic risk, its relationship with metabolic dysfunction is heterogeneous and context-dependent. Cardiometabolic risk in psoriasis arises from multiple interacting but partially independent pathways, including adipose tissue dysfunction, insulin resistance, endothelial injury, and immune activation [19]. These processes do not necessarily evolve in parallel, particularly in cross-sectional clinical data from patients with high comorbidity burden [3,20].

The present findings support a model of partial independence between inflammatory and metabolic processes, without implying a direct causal relationship between these domains. Acute-phase markers such as CRP and ESR showed associations with metabolic indices in univariate analyses, but these relationships did not persist after multivariable adjustment. This suggests that systemic inflammation may reflect overall disease complexity rather than acting as an independent determinant of metabolic syndrome in all patients with psoriasis [19,21].

The independent association observed between ESR and insulin resistance may reflect its ability to capture cumulative inflammatory exposure rather than short-term fluctuations, suggesting that markers of chronic inflammation may have distinct metabolic implications compared with acute-phase responses.

PCA further supports this interpretation by demonstrating a clear separation between inflammatory and metabolic domains. Inflammatory markers clustered within an immune–inflammatory axis, whereas TyG, AIP, and METS-IR defined a distinct metabolic–insulin resistance axis. This separation reflects the expected clustering of inflammatory and metabolic variables and supports the structural organization of these biomarker domains within the dataset rather than indicating a novel biological discovery. Similar multidimensional analyses have reported comparable clustering patterns, supporting the conceptual distinction between inflammatory and metabolic components of psoriatic disease [22]. Accordingly, PCA in this context serves primarily as a confirmatory tool that reinforces the internal consistency of the analytical framework.

Adipose tissue inflammation, hepatic metabolic regulation, endothelial dysfunction, and immune signaling operate through interconnected but non-identical systems that collectively contribute to cardiometabolic risk in psoriasis [3,21]. Importantly, the relationship between inflammation and insulin resistance is not uniform and may be strongly influenced by the distribution and functional characteristics of adipose tissue. Visceral (central) adiposity is metabolically more active than subcutaneous fat and is associated with increased release of pro-inflammatory adipokines [23], enhanced lipotoxicity and metabolic stress [24], and greater impairment of insulin signaling pathways. In contrast, more diffuse adiposity may exert a comparatively weaker effect on systemic metabolic and inflammatory profiles, reflecting differences in adipose tissue inflammatory activity and endocrine function [25]. These distinctions suggest that the observed associations between inflammatory and metabolic markers in psoriasis may reflect underlying differences in adipose tissue distribution rather than a direct causal relationship between inflammation and insulin resistance.

Visceral adiposity further promotes insulin resistance through lipotoxicity, ectopic lipid deposition, and activation of intracellular stress pathways, including JNK and NF-κB signaling, which interfere with insulin signaling and glucose metabolism [24,26,27]. These mechanisms provide a biologically plausible framework that may help explain the stronger association of TyG and METS-IR with metabolic syndrome observed in the present study, although causal relationships cannot be established.

Insulin resistance and lipid dysregulation may be driven primarily by adipose-derived inflammatory mediators and visceral-adiposity-related metabolic stress rather than circulating acute-phase reactants alone. This reflects the endocrine and immunologic activity of metabolically active adipose depots [28].

Adipokine imbalance further supports this interpretation. Elevated leptin and resistin levels, together with reduced adiponectin, contribute to insulin resistance and metabolic syndrome in psoriasis [29,30]. These mechanisms may explain why metabolic indices show stronger associations with metabolic syndrome than systemic inflammatory markers in cross-sectional analyses [22].

The prominent role of insulin-resistance-related indices in the present study is therefore noteworthy. The TyG index remained the strongest independent predictor of metabolic syndrome after adjustment for inflammatory markers, highlighting the importance of metabolic regulatory dysfunction in cardiometabolic risk among patients with psoriasis. These findings should be interpreted as associative rather than causal, given the observational nature of the study. Insulin resistance has been consistently linked to cardiovascular disease, non-alcoholic fatty liver disease, and metabolic syndrome in psoriatic populations [13,19].

The observed interaction between adiposity and insulin resistance suggests that metabolic risk amplification is not merely additive but may be synergistic. Increasing adipose tissue mass promotes adipokine dysregulation, macrophage infiltration, and low-grade inflammation within visceral depots, thereby intensifying insulin signaling impairment. This may explain the stronger effect of TyG at higher BMI levels, reflecting a threshold-dependent increase in cardiometabolic risk [23,25]. Evidence from population-based and cohort studies supports the clinical relevance of TyG in psoriasis. Associations have been reported with disease presence, cardiovascular risk, subclinical atherosclerosis, and adverse cardiovascular outcomes, including longitudinal risk prediction in high-risk populations [15,31,32,33]. Although TyG and METS-IR are practical and widely used surrogate indices of insulin resistance, they do not directly measure insulin sensitivity and have not been consistently validated against gold-standard techniques such as the hyperinsulinemic–euglycemic clamp or HOMA-IR in all populations. Their values may also vary depending on demographic, metabolic, and ethnic characteristics. Therefore, these indices should be interpreted as indirect markers of metabolic dysfunction rather than definitive measures of insulin resistance, particularly in heterogeneous clinical populations.

Additional evidence supports the role of metabolic regulatory dysfunction. METS-IR has been associated with psoriasis prevalence in population-level analyses, suggesting that metabolic impairment may be intrinsically linked to psoriatic disease even in the absence of overt inflammatory exacerbation [34]. Similarly, metabolic complications such as fatty liver disease appear more closely related to insulin resistance than to systemic inflammatory markers [35]. These findings are consistent with the present results, in which metabolic indices were the main independent discriminators of metabolic syndrome.

The differential behavior of inflammatory biomarkers also warrants consideration. Acute-phase reactants showed consistent associations with metabolic indices, whereas hematologic markers such as NLR and SII demonstrated limited relationships. This likely reflects biological specificity, as CRP and ESR integrate signals from multiple systemic processes, including metabolic stress, vascular injury, and comorbidity burden [13,36].

In contrast, CBC-derived immune ratios primarily reflect leukocyte dynamics and innate immune activation. They serve as indicators of inflammatory activity rather than direct mediators of metabolic dysfunction. Although useful for assessing inflammatory burden, their relationship with metabolic alterations appears indirect and context-dependent [37].

The clinical composition of the cohort likely influenced these findings. Because most patients were hospitalized for non-dermatologic conditions, the study population represents individuals with substantial systemic comorbidity. Hospital-based psoriasis cohorts are known to exhibit higher prevalence of metabolic syndrome, cardiovascular disease, and multi-organ involvement compared with outpatient populations [16].

Treatment-related factors may also influence these relationships. Systemic therapies, particularly biologic agents, may modify metabolic parameters and insulin resistance, although available evidence remains heterogeneous [38,39]. These findings highlight the dynamic interplay between inflammatory control and metabolic regulation and suggest that metabolic risk cannot be inferred solely from inflammatory activity.

Several limitations should be acknowledged. The retrospective cross-sectional design precludes causal inference and does not allow evaluation of temporal relationships between inflammation and metabolic dysfunction; therefore, the observed associations should be interpreted as non-directional rather than causal.

The hospital-based nature of the cohort introduces potential selection bias and limits external validity. Because most patients had psoriasis recorded as a secondary diagnosis, the study population likely reflects individuals with substantial comorbidity burden. Consequently, the observed associations may represent a comorbidity-driven phenotype rather than psoriasis-specific mechanisms. Although information on primary versus secondary diagnosis was available, the relatively small number of patients with psoriasis as a primary diagnosis precluded adequately powered subgroup analyses.

Dermatological characterization was also incomplete. Although most patients had plaque psoriasis, detailed phenotypic classification was not consistently available in the medical records and could not be systematically analyzed, potentially contributing to heterogeneity in inflammatory and metabolic profiles. In addition, standardized measures of disease severity, such as PASI, were not consistently available, limiting adjustment for disease activity. Treatment-related variables represent another source of uncertainty. While treatment data were available, they were heterogeneous and not standardized in terms of duration, dosing, or prior exposure, which limited their inclusion in multivariable analyses. Importantly, systemic and biologic therapies, as well as underlying disease activity, may significantly influence both inflammatory activity and metabolic parameters. Therefore, variability in treatment exposure and the lack of standardized assessment of disease activity may have affected the observed levels of inflammatory biomarkers (e.g., CRP, ESR, NLR, SII) as well as metabolic indices, introducing potential residual confounding. Similarly, important confounding factors, including concomitant medications (such as lipid-lowering and antidiabetic therapies) and lifestyle factors like diet and physical activity, were not consistently available and could not be accounted for, raising the possibility of residual confounding.

Further limitations include the lack of detailed characterization of diabetes (e.g., insulin dependence or treatment status), which may have influenced the assessment of metabolic status. In addition, the study period overlapped with the COVID-19 pandemic, which may have affected metabolic parameters, inflammatory profiles, and healthcare utilization. Because detailed data on SARS-CoV-2 infection and related factors were not consistently available, their potential impact could not be specifically evaluated.

Taken together, these limitations indicate that the findings should be interpreted with caution and considered hypothesis-generating rather than definitive.

Despite these constraints, the study has important strengths. The integration of correlation analysis, multivariable regression, and principal component analysis enabled simultaneous evaluation of association strength, independent predictive value, and latent biological structure. The use of composite metabolic indices provided refined characterization of insulin resistance and cardiometabolic risk beyond conventional laboratory measures. This multidimensional analytical framework aligns with current priorities in systemic disease characterization and precision risk stratification.

From a clinical perspective, the results suggest that metabolic risk assessment in psoriasis may benefit from incorporating insulin-resistance-related indices alongside traditional inflammatory markers. Although systemic inflammation is a defining feature of psoriatic disease, it did not independently discriminate metabolic syndrome in this cohort. At the same time, the moderate predictive performance of metabolic indices underscores the need for comprehensive assessment rather than reliance on single biomarkers. These findings support emerging recommendations advocating structured cardiometabolic screening strategies that integrate metabolic indices, anthropometric measures, and cardiovascular risk profiling rather than reliance solely on inflammatory parameters [17].

Future longitudinal studies are needed to clarify the temporal and potentially causal relationships between inflammatory activity, insulin resistance, and cardiometabolic outcomes in psoriasis. Prospective investigations evaluating whether modification of inflammatory burden or metabolic risk results in measurable improvement in systemic outcomes would provide important mechanistic insight. In addition, validation of composite metabolic indices across diverse clinical populations, combined with imaging-based vascular assessment and treatment stratification, may further refine cardiometabolic risk prediction and support translation of biomarker research into clinical practice.

## 4. Materials and Methods

### 4.1. Study Design and Population

This retrospective observational study included adult patients (≥18 years) diagnosed with psoriasis who were hospitalized between January 2020 and December 2022 at Bihor County Emergency Clinical Hospital, Oradea, Romania. Patients were identified from the institutional electronic medical record system irrespective of the admitting department. The diagnosis of psoriasis was confirmed by a board-certified dermatologist based on established clinical criteria. Both patients hospitalized with psoriasis as a primary diagnosis and those with psoriasis recorded as a secondary diagnosis were eligible, provided complete clinical and laboratory data were available.

Inclusion criteria were: confirmed diagnosis of psoriasis; age ≥ 18 years; and availability of complete inflammatory and metabolic laboratory parameters obtained at hospital admission prior to therapeutic intervention. Exclusion criteria comprised active infection, malignancy under active treatment, acute cardiovascular events during hospitalization, advanced hepatic, renal, or cardiac failure, missing key laboratory variables, and duplicate records.

A total of 235 patients met the eligibility criteria and were included in the final analysis. Clinical, laboratory, and hospitalization data were retrieved from the institutional electronic medical record system. The study period overlapped with the COVID-19 pandemic; however, specific data regarding SARS-CoV-2 infection status, pandemic-related behavioral changes, or treatment interruptions were not consistently available in the electronic medical records and were therefore not included in the analysis. All patients provided written informed consent at the time of hospitalization, allowing the use of their anonymized clinical and laboratory data for research purposes in accordance with institutional regulations.

### 4.2. Clinical and Demographic Variables

Demographic variables included age, sex, area of residence (urban/rural), educational level, and disease duration (years since diagnosis). Clinical data extracted from medical records included psoriasis clinical form and treatment modality (topical therapy, systemic oral therapy, biologic therapy, or phototherapy). Information on disease severity (Psoriasis Area and Severity Index, PASI) and quality of life (Dermatology Life Quality Index, DLQI) was available only for a limited subset of patients and was therefore not included in the analysis. For analytical purposes, patients were grouped into plaque psoriasis and other forms, due to the small number of cases in specific subtypes and heterogeneous reporting.

Comorbidities were recorded as binary variables (present/absent) and included arterial hypertension, dyslipidemia, type 2 diabetes mellitus, hepatic disorders, renal disease, pulmonary disease, cardiovascular disease, autoimmune disorders, and psychiatric conditions. Lifestyle factors included smoking status and alcohol consumption. Body mass index (BMI) was calculated as weight (kg)/height^2^ (m^2^) and categorized according to WHO criteria.

### 4.3. Inflammatory and Metabolic Biomarkers

Systemic inflammatory and metabolic status was evaluated using conventional laboratory parameters and composite indices reflecting immune activation and cardiometabolic dysregulation. All biochemical parameters used for metabolic indices (including glucose and lipid profile) were obtained from fasting blood samples, according to standard hospital protocols.

Inflammatory markers included C-reactive protein (CRP), erythrocyte sedimentation rate (ESR), neutrophil-to-lymphocyte ratio (NLR), and the systemic immune–inflammation index (SII). Metabolic evaluation included fasting plasma glucose, lipid profile (total cholesterol, HDL, LDL, triglycerides), and derived indices of insulin resistance and atherogenic risk, namely the Atherogenic Index of Plasma (AIP), Triglyceride–Glucose index (TyG), and Metabolic Score for Insulin Resistance (METS-IR).

Definitions, formulas, units of measurement, and corresponding pathophysiological axes for all biomarkers included in the integrative analysis are summarized in Table 8.

Metabolic syndrome was defined according to the revised NCEP ATP III criteria as the presence of at least three of the following components: (1) abdominal obesity (BMI ≥ 30 kg/m^2^, used as a surrogate for central obesity in the absence of waist circumference data); (2) elevated triglycerides (≥150 mg/dL); (3) reduced HDL cholesterol (<40 mg/dL in men, <50 mg/dL in women); (4) elevated blood pressure (≥130/85 mmHg or use of antihypertensive medication); and (5) elevated fasting glucose (≥100 mg/dL or diagnosis of diabetes mellitus) [49].

### 4.4. Outcomes and Statistical Analysis

The primary outcome was the presence of metabolic syndrome (MetS), defined according to the revised NCEP ATP III criteria as the presence of at least three abnormal metabolic components. Secondary outcomes included (i) evaluation of associations between inflammatory and metabolic biomarkers and (ii) identification of latent inflammatory–metabolic domains using principal component analysis (PCA).

Statistical analyses were performed using JASP (version 0.19.3) with standard default settings. All tests were two-tailed, and a *p*-value < 0.05 was considered statistically significant. Continuous variables were assessed for normality using the Shapiro–Wilk test and are presented as mean ± standard deviation or median (interquartile range), as appropriate. Categorical variables are expressed as frequencies and percentages.

Group comparisons were performed using the independent samples *t*-test or Mann–Whitney U test for continuous variables and the chi-square test for categorical variables, as appropriate. Spearman correlation analysis was used to evaluate associations between inflammatory and metabolic indices.

To identify independent predictors of MetS, multivariable logistic regression models were constructed. Age and body mass index (BMI) were included as clinical covariates. The triglyceride–glucose (TyG) index was evaluated as a marker of insulin resistance. All predictors were entered simultaneously using the enter method without stepwise selection. Odds ratios (ORs) with 95% confidence intervals (CIs) were reported.

To assess the potential synergistic effect between adiposity and insulin resistance, BMI and TyG were mean-centered (BMI_c and TyG_c), and an interaction term (BMI_c × TyG_c) was introduced into the regression model.

Model discrimination was evaluated using the area under the receiver operating characteristic curve (AUC). Classification performance was assessed using sensitivity, specificity, and overall accuracy. Multicollinearity was examined using variance inflation factors (VIFs), with VIFs < 5 considered acceptable.

To evaluate independent determinants of insulin resistance, multivariable linear regression models were constructed using METS-IR as the dependent variable. Standardized beta coefficients (β) were reported to compare relative effect sizes across predictors.

Internal validation of regression coefficients was performed using bootstrap resampling (1000 iterations) with bias-corrected accelerated confidence intervals. Principal component analysis (PCA) was performed on seven biomarkers (NLR, SII, CRP, ESR, TyG, AIP, METS-IR) using the correlation matrix with Varimax rotation. Sampling adequacy was assessed using the Kaiser–Meyer–Olkin (KMO) index and Bartlett’s test of sphericity. Components with eigenvalues > 1 were retained. All variables were standardized prior to PCA to ensure comparability of scales.

Cases with missing key laboratory variables were excluded prior to analysis, and no data imputation was performed.

The study protocol was approved by the Ethics Committee of Bihor County Emergency Clinical Hospital, Oradea, Romania (Approval No. 31133/11 October 2024). All procedures complied with the Declaration of Helsinki.

## 5. Conclusions

In this hospitalized cohort of patients with psoriasis, inflammatory and metabolic biomarkers clustered into two distinct domains, consistent with the expected separation between inflammatory and insulin-resistance-related processes. Although inflammatory markers showed modest associations with metabolic indices, they did not independently discriminate metabolic syndrome in multivariable models.

In contrast, the TyG index showed the strongest independent association with metabolic syndrome, highlighting the potential relevance of insulin-resistance-related metabolic dysfunction in this population. These findings support a multidimensional model of psoriatic disease in which inflammatory and metabolic processes coexist but contribute differently to cardiometabolic risk.

From a clinical perspective, these results suggest that metabolic risk assessment in psoriasis may benefit from incorporating insulin-resistance-related indices alongside traditional inflammatory markers. However, given the cross-sectional design, these findings should be interpreted as associative rather than causal, and prospective studies are needed to clarify the temporal relationships between inflammation, insulin resistance, and cardiometabolic outcomes, particularly as the present results reflect a hospitalized, multimorbid population with high cardiometabolic burden and may not be directly generalizable to routine outpatient psoriasis cohorts.

## Figures and Tables

**Table 1:** Baseline characteristics and healthcare utilization of the study population (*N* = 235).

Variable	Value
A. Sociodemographic characteristics	
Age (years), median (IQR)	58 (19)
Gender, *n* (%)	Male: 125 (53.2%) Female: 110 (46.8%)
Residence, *n* (%)	Urban: 119 (50.6%) Rural: 116 (49.4%)
B. Clinical and metabolic characteristics	
Disease duration (years), median (IQR)	10 (12)
Psoriasis type, *n* (%)	Plaque psoriasis: 125 (53.2%) Other forms: 110 (46.8%)
Psoriatic arthritis, *n* (%)	35 (14.9%)
Treatment status, *n* (%)	No treatment: 101 (43.0%) Topical therapy: 97 (41.3%) Systemic therapy: 61 (26.0%) Biologic therapy: 10 (4.3%) Phototherapy: 11 (4.7%)
BMI (kg/m^2^), median (IQR)	28.9 (7.2)
Nutritional status, *n* (%)	Underweight: 6 (2.6%) Normal weight: 34 (14.5%) Overweight: 99 (42.1%) Obesity class I: 53 (22.6%) Obesity class II: 18 (7.7%) Obesity class III: 25 (10.6%)
Metabolic syndrome, *n* (%)	91 (38.7%)
C. Comorbidities, *n* (%)	
Hypertension	146 (62.1%)
Dyslipidemia	83 (35.3%)
Diabetes mellitus	65 (27.7%)
Other cardiovascular disease	75 (31.9%)
Pulmonary disease	39 (16.6%)
Hepatic disease	58 (24.7%)
Renal disease	28 (11.9%)
Psychiatric disorders	23 (9.8%)
D. Healthcare utilization	
Days of hospitalization, median (IQR)	5 (4)
Number of hospitalizations, median (IQR)	1 (0)
Distribution of hospitalizations, *n* (%)	1 admission: 207 (88.1%) 2 admissions: 20 (8.5%) ≥3 admissions: 8 (3.4%)
Diagnosis type, *n* (%)	Primary: 23 (9.8%) Secondary: 212 (90.2%)

BMI, body mass index; IQR, interquartile range; *N*, total number of participants. Treatment categories are not mutually exclusive.

**Table 2:** Systemic inflammatory indices and inflammation severity.

Inflammatory Index	Value
Neutrophils (×10^3^/µL), median (IQR)	5.17 (3.30)
Lymphocytes (×10^3^/µL), median (IQR)	1.89 (1.21)
Platelets (×10^3^/µL), median (IQR)	248.5 (98.0)
NLR, median (IQR)	2.556 (2.221)
SII, median (IQR)	668.96 (631.06)
CRP (mg/L), median (IQR)	6.8 (11.9)
ESR (mm/h), median (IQR)	21.0 (20.5)
CRP elevated, *n* (%)	137 (58.3%)
ESR elevated, *n* (%)	157 (66.8%)
Inflammation severity (SII categories), *n* (%)	Mild 77 (32.8%) Moderate 63 (26.8%) Severe 95 (40.4%)

CRP, C-reactive protein; ESR, erythrocyte sedimentation rate; IQR, interquartile range; NLR, neutrophil-to-lymphocyte ratio; SII, systemic immune–inflammation index.

**Table 3:** Metabolic indices and metabolic comorbidity burden (*N* = 235).

Metabolic Index	Value
Glucose (mg/dL), median (IQR)	100 (36)
Total cholesterol (mg/dL), median (IQR)	181 (40)
HDL cholesterol (mg/dL), median (IQR)	38 (18)
LDL cholesterol (mg/dL), median (IQR)	105 (59.5)
Triglycerides (mg/dL), median (IQR)	116 (72.5)
TyG index, median (IQR)	8.751 (0.744)
AIP, median (IQR)	0.486 (0.411)
METS-IR, median (IQR)	39.420 (15.439)
Hyperglycemia, *n* (%)	53 (22.6%)
Hypercholesterolemia, *n* (%)	83 (35.3%)
Dyslipidemia, *n* (%)	83 (35.3%)
Obesity (BMI-based), *n* (%)	96 (40.8%)
Metabolic syndrome, *n* (%)	91 (38.7%)

AIP, atherogenic index of plasma; BMI, body mass index; HDL, high-density lipoprotein; IQR, interquartile range; LDL, low-density lipoprotein; METS-IR, metabolic score for insulin resistance; TyG, triglyceride–glucose index.

**Table 4:** Comparison of inflammatory and metabolic indices between patients with and without metabolic syndrome (Mann–Whitney U test).

Marker	SM− Median (Q1–Q3)	SM+ Median (Q1–Q3)	U	*p*-Value	Effect Size (r)
SII	668.96 (426.01–1037.56)	676.20 (394.15–974.67)	6527.0	0.962	−0.004
NLR	2.56 (1.93–4.10)	2.82 (1.77–4.19)	6601.5	0.923	0.008
CRP	6.45 (2.86–13.90)	8.00 (3.55–16.25)	5432.5	0.119	−0.122
ESR	18.00 (12.00–31.00)	23.00 (14.50–42.00)	5605.0	0.062	−0.145
TyG	8.53 (8.19–8.88)	9.05 (8.71–9.42)	3053.5	<0.001	−0.534
AIP	0.42 (0.24–0.58)	0.61 (0.44–0.81)	4081.5	<0.001	−0.377
METS-IR	36.13 (30.80–42.30)	47.24 (39.18–55.68)	3196.0	<0.001	−0.512

AIP, atherogenic index of plasma; CRP, C-reactive protein; ESR, erythrocyte sedimentation rate; METS-IR, metabolic score for insulin resistance; NLR, neutrophil-to-lymphocyte ratio; SII, systemic immune–inflammation index; SM−, without metabolic syndrome; SM+, with metabolic syndrome; TyG, triglyceride–glucose index; U, Mann–Whitney U statistic.

**Table 5:** Spearman correlations between inflammatory and metabolic indices.

Inflammatory Index	TyG	AIP	METS-IR	TG
NLR	0.118	0.023	0.026	−0.037
SII	0.172 **	0.026	0.026	0.052
CRP	0.339 ***	0.284 ***	0.242 ***	0.239 ***
ESR	0.270 ***	0.241 ***	0.256 ***	0.206 **

AIP, atherogenic index of plasma; CRP, C-reactive protein; ESR, erythrocyte sedimentation rate; METS-IR, metabolic score for insulin resistance; NLR, neutrophil-to-lymphocyte ratio; SII, systemic immune–inflammation index; TG, triglycerides; TyG, triglyceride–glucose index; Statistical significance levels: ** *p* < 0.01, *** *p* < 0.001.

**Table 6:** Multivariable linear regression model evaluating inflammatory predictors of METS-IR.

Predictor	B (SE)	β Standardized	t	*p*-Value	95% CI
CRP	0.019 (0.034)	0.047	0.544	0.587	−0.049 to 0.086
ESR	0.102 (0.042)	0.214	2.433	0.016	0.019 to 0.184
NLR	0.094 (0.360)	0.038	0.260	0.795	−0.616 to 0.803
SII	−0.001 (0.001)	−0.156	−1.086	0.279	−0.003 to 0.0009

Model R^2^ = 0.054; Adjusted R^2^ = 0.037; F = 3.152; *p* = 0.015. B, unstandardized regression coefficient; SE, standard error; β, standardized regression coefficient; CI, confidence interval; CRP, C-reactive protein; ESR, erythrocyte sedimentation rate; METS-IR, metabolic score for insulin resistance; NLR, neutrophil-to-lymphocyte ratio; SII, systemic immune–inflammation index; R^2^, coefficient of determination.

**Table 7:** Multivariable logistic regression model predicting metabolic syndrome.

Predictor	OR	95% CI	*p*-Value
Age (per year)	1.046	1.021–1.071	<0.001
BMI_c	1.092	1.033–1.154	0.002
TyG_c	5.15	2.56–10.34	<0.001
BMI_c × TyG_c	1.21	1.07–1.36	0.002

BMI_c and TyG_c represent mean-centered variables. The interaction term reflects the interaction between adiposity and insulin-resistance-related indices.

**Table 8:** Inflammatory and metabolic biomarkers included in the integrative analysis.

Category	Biomarker	Formula/Unit	Reference Interval (Adults)	Pathophysiological Axis
Inflammatory	CRP	mg/L	<5 mg/L (normal); 5–10 mg/L (mild elevation); >10 mg/L (significant inflammation)	Hepatic acute-phase response
	ESR	mm/h	Men <15 mm/h; women <20 mm/h (age-adjusted upper limit: age/2 for men; (age + 10)/2 for women)	Chronic inflammatory activity
	NLR	Neutrophils/Lymphocytes	0.8–3.0 (physiological range); >3.0–5.0 (elevated inflammatory activity)	Innate immune activation [40,41]
	SII	Neutrophils × Platelets/Lymphocytes	300–800 (reference range); >800–1000 (elevated systemic inflammation)	Platelet–immune interaction [42,43]
Metabolic	Fasting glucose	mg/dL	70–99 (normal); 100–125 (impaired fasting glucose); ≥126 (diabetes)	Glycemic control
	Triglycerides	mg/dL	<150 (normal); 150–199 (borderline); 200–499 (high); ≥500 (very high)	Lipid metabolism
	HDL cholesterol	mg/dL	Men ≥40; women ≥50; <40 low cardioprotective effect	Reverse cholesterol transport
	AIP	log10 (TG/HDL)	<0.11 (low risk); 0.11–0.21 (intermediate risk); >0.21 (high atherogenic risk)	Atherogenic risk [44,45]
	TyG	ln (TG × Glucose/2)	7.5–8.5 (reference range); ≥8.8–9.0 (suggestive of insulin resistance)	Insulin resistance surrogate [46,47]
	METS-IR	ln[(2 × Glucose) + Triglycerides] × BMI/ln(HDL)	<35 (low IR); 35–45 (intermediate IR); >45 (elevated IR)	Insulin resistance composite [46,48]

CRP, C-reactive protein; ESR, erythrocyte sedimentation rate; NLR, neutrophil-to-lymphocyte ratio; SII, systemic immune–inflammation index; HDL, high-density lipoprotein cholesterol; TG, triglycerides; AIP, atherogenic index of plasma; TyG, triglyceride–glucose index; METS-IR, metabolic score for insulin resistance; BMI, body mass index; ln, natural logarithm; log10, base-10 logarithm.

## Data Availability

More data are available per request at the first author.

## Abbreviations

The following abbreviations are used in this manuscript:

AHA	American Heart Association
AIC	Akaike information criterion
AIP	Atherogenic index of plasma
ATP III	Adult treatment panel III
AUC	Area under the curve
BMI	Body mass index
CBC	Complete blood count
CRP	C-reactive protein
CVD	Cardiovascular disease
DLQI	Dermatology life quality index
ESR	Erythrocyte sedimentation rate
FPG	Fasting plasma glucose
GPA	Global Psoriasis Atlas
HDL	High-density lipoprotein
IL-17	Interleukin 17
IL-23	Interleukin 23
IL-6	Interleukin 6
IQR	Interquartile range
KMO	Kaiser–Meyer–Olkin index
LDL	Low-density lipoprotein
METS-IR	Metabolic score for insulin resistance
MetS	Metabolic syndrome
NCEP	National Cholesterol Education Program
NHLBI	National Heart, Lung, And Blood Institute
NLR	Neutrophil-to-lymphocyte ratio
OR	Odds ratio
PASI	Psoriasis area and severity index
PCA	Principal component analysis
ROS	Reactive oxygen species
SD	Standard deviation
SII	Systemic immune–inflammation index
TG	Triglycerides
TNF-α	Tumor necrosis factor alpha
TyG	Triglyceride–glucose index
VIF	Variance inflation factor
WHO	World Health Organization
